# Lactoferrin Metal Saturation—Which Form Is the Best for Neonatal Nutrition?

**DOI:** 10.3390/nu12113340

**Published:** 2020-10-30

**Authors:** Grzegorz Majka, Magdalena Pilarczyk-Zurek, Agnieszka Baranowska, Beata Skowron, Magdalena Strus

**Affiliations:** 1 Chair of Microbiology, Faculty of Medicine, Jagiellonian University Medical College, Czysta 18, 31-121 Kraków, Poland; grzegorz.majka@uj.edu.pl (G.M.); magdalena.pilarczyk-zurek@uj.edu.pl (M.P.-Z.); 2 Chair of Immunology, Faculty of Medicine, Jagiellonian University Medical College, 31-121 Kraków, Poland; 3 Department of Microbiology, Faculty of Biochemistry, Biophysics and Biotechnology, Jagiellonian University, 30-387 Kraków, Poland; 4 Chair of Pathophysiology, Faculty of Medicine, Jagiellonian University Medical College, 31-121 Kraków, Poland; agnieszka.1.baranowska@uj.edu.pl (A.B.); beata.skowron@onkologia.krakow.pl (B.S.); 5 Department of Clinical Biochemistry, Center of Oncology—Maria Skłodowska-Curie Memorial Institute, Cracow Division, 31-115 Kraków, Poland

**Keywords:** lactoferrin, bacterial translocation, iron, manganese, infection, *Escherichia coli*

## Abstract

We evaluated the impact of metal saturation of lactoferrin (with iron and manganese) on population numbers of pathogenic species relevant for neonatal sepsis that commonly originates from the gut due to bacterial translocation. Little attention has been paid to how metal ions bound to the protein affect its activity. Several reference and clinical strains as well as probiotic strains were incubated with different forms of lactoferrin: metal-depleted (apolactoferrin), iron-saturated (hololactoferrin) and manganese-saturated lactoferrin. We also attempted to confirm the observed effects of lactoferrin forms in vivo using rat pups. The observed decrease in population numbers of Gram-negative rods could not be confirmed by quantitative plating—lactoferrin may regulate these populations diversely (e.g., by anti-biofilm activity) and contribute to the inhibition of inflammatory response. We did not see any effect of lactoferrin forms on staphylococci and bifidobacteria. However, we have noted a significant increase of population numbers of *Lactobacillus* strains upon incubation with manganese-saturated lactoferrin. These results were confirmed in vivo in a rat model. Metal saturation is an underestimated factor regulating lactoferrin activity. Some forms are more potent in the inhibition of pathogenic species while others, such as manganese-saturated lactoferrin, could contribute to the restoration of gut homeostasis.

## 1. Introduction

The colostrum and breast milk of mammals contain extremely important nutrients for infants in the first days after delivery. They contain multiple protective peptides and proteins including an iron-binding glycoprotein, lactoferrin (Lf) The concentration of Lf is 5–13 g/L in the colostrum and 1.5–4.5 g/L in milk [1,2]. It is plausible that this protein plays an important role during neonatal development as the daily supply of Lf in an infant ranges between 0.4 g and 1.2 g/kg body weight [3].

Scientists have been developing the idea that Lf is able to modulate the newborn gastrointestinal tract microbiota composition thanks to bactericidal and bacteriostatic properties [4]. Furthermore, in vitro studies have proved lactoferrin’s antioxidant activity, the potential to inhibit bacterial biofilm formation and the regulation of immune cells’ response to the lipopolysaccharide (LPS), the endotoxin of Gram-negative bacteria [5].

Lactoferrin is characterized by high stability. The release of iron requires the destabilization of the protein structure, e.g., by lowering the pH. This phenomenon of ferric ion release can occur in the upper gastrointestinal tract of the newborn under the influence of a gastric pH equal to 4.0 [5,6]. Thus, both a metal-free form apolactoferrin (apoLf) and an iron-saturated hololactoferrin (holoLf) can exist in the newborn’s gastrointestinal tract at the same time.

It is important to emphasize that every supply of free ferric ions in a gastrointestinal tract may stimulate the overgrowth of those Gram-negative rods that produce siderophores, which are low molecular weight substances (but also proteins) that capture ferric ions and fuel the rapid multiplication of Gram-negative bacteria [7]. Even a small increase in the *Escherichia coli* population in the very low birth weight (VLBW, i.e., below 1500 g) newborn’s gastrointestinal tract can impair the mucosal barrier function and lead to the translocation of bacteria, primarily Gram-negative pathogenic flora, from the intestinal lumen into the blood system, leading to sepsis. In newborns, sepsis is a major cause of morbidity and mortality.

It is possible that different forms of lactoferrin (metal-depleted apolactoferrin, iron-saturated hololactoferrin or manganese-saturated lactoferrin) may be recognized as the key factors affecting the possibility of *E. coli* translocation from the gut.

Recently, we have observed a growing interest in clinical applications of lactoferrin to improve the intestinal barrier function in preterm infants providing protection against translocation, sepsis and necrotizing enterocolitis.

The first results of the clinical trials conducted about 10 years ago were very promising; it was proved that an oral supplementation of Lf significantly reduced the number of episodes of late-onset sepsis in newborns with a VLBW [7]. However, in recent publications authors have begun to doubt whether lactoferrin really reduces the episodes of sepsis in newborns. They emphasize the fact that the sample sizes are too small and they conclude that oral supplementation with bovine lactoferrin does not reduce the incidence of sepsis in infants with birth weights lower than 2000 g [8].

Why are the results of clinical trials so different? As very little is known about how metal saturation of this protein may modify the composition of the newborn’s microbiota, it is probably a great opportunity to return to basic studies on lactoferrin.

Therefore, the main objective of this study was to elucidate how iron-depleted, iron-saturated and manganese-saturated forms of lactoferrin can regulate the populations Gram-negative and Gram-positive bacteria (including *Lactobacillus* and *Bifidobacterium* strains) colonizing the newborn’s gastrointestinal tract a few hours after delivery.

We also studied the influence of various lactoferrin forms on the gut microbiota by using prematurely weaned rat pups; namely, levels of potentially pathogenic *E. coli* as well as probiotic strains of the *Lactobacillus* genus. The use of an animal model allowed us to observe the effect of lactoferrin forms and saturation levels in a mammalian system similar to those found in human newborns especially those with a VLBW that are separated from their mothers and are at a particularly high risk of developing sepsis.

## 2. Materials and Methods

### 2.1. Forms of Lactoferrin Used in Experiments

Bovine lactoferrin was purchased from FrieslandCampina (Amersfoort, The Netherlands) and exhibited iron saturation of 10.2%. The modified forms (iron-depleted (apolactoferrin, apoLf, 1.2 ± 0.2% Fe saturation), iron-saturated (hololactoferrin, holoLf, 71.8 ± 6.5% Fe saturation) and manganese-saturated lactoferrin (MnLf, 1.2 ± 0.2% Fe saturation, 47.1 ± 2.0% Mn^3+^ saturation)) were prepared as described before [9,10].

In further experiments, all forms of lactoferrin were tested at concentrations 0.6, 5 and 40 mg/mL. High concentrations of lactoferrin have been described as bactericidal/bacteriostatic and have been used in dietary supplements intended for newborns and children [11].

### 2.2. Pathogenic Gram-Negative and Gram-Positive Bacterial Strains and Growth Conditions

The experiments involved the Gram-negative reference bacterial species *Escherichia coli* (ATCC 25922), *Klebsiella pneumoniae* (ATCC 700603) and the Gram-positive reference strain *Staphylococcus aureus* (ATCC 25923). Pathogenic Gram-negative species tested included clinical strains of *Escherichia coli* (CM226), *Klebsiella pneumoniae* (CM18) and a Gram-positive strain *Staphylococcus aureus* (CM30). Pathogenic strains were isolated from the blood of very low birth weight neonates with clinical symptoms of sepsis confirmed by blood culture. Neonates were treated in Polish hospitals within The Polish Neonatology Surveillance Network (PNSN). Utilization of the data collected in the PNSN for scientific purposes was approved by the Bioethics Committee of Jagiellonian University Medical College (no. KBET/221/B/2011).

*E. coli* and *K. pneumoniae* strains were passaged on MacConkey Agar (Oxoid, UK) and *S. aureus* on Blood Agar (Oxoid, UK) under aerobic conditions at 37 °C. Before the experiment, all strains were cultured in liquid TSB (tryptic soy broth, Oxoid, UK) overnight.

### 2.3. Probiotic Bacteria Strains and Growth Conditions

The experiments involved reference probiotic bacterial strains *Lactobacillus plantarum* (ATCC 14431), *Lactobacillus rhamnosus* (ATCC 53103), *Bifidobacterium breve* (ATCC 15700) and *Bifidobacterium longum* (ATCC 15707).

Investigated probiotic bacteria species included strains isolated from commercially available probiotic formulae (Lactoral^®^ and FFbaby^®^, Biomed, Poland) *L. plantarum* (PL02), *L. rhamnosus* (KL53A), *B. breve* (PB04) and *B. longum* (PL03).

*Lactobacillus* strains were passaged on de Man, Rogosa and Sharpe (MRS) agar (Oxoid, UK) and cultured in MRS broth (Oxoid, UK). *Bifidobacterium* strains were passaged on trypticase-phytone-yeast agar (TPY, Fluka, Switzerland) and cultured in liquid TPY broth (Fluka, Switzerland). *Lactobacillus* and *Bifidobacterium* strains were cultured under anaerobic conditions at 37 °C. Before the experiment, strains were cultured for 48 h in liquid broth.

### 2.4. The Effect of Various Forms of Lactoferrin on Pathogenic and Probiotic Bacterial Growth

The cultures of Gram-negative rods *E. coli* and *K. pneumoniae* were performed in a liquid M9 medium (Sigma-Aldrich, St. Louis, MO, USA), a broth with a defined minimal composition. To observe bacterial growth, it was necessary to add ferric salt 0.2 mM (FeSO_4_)·7H_2_O to achieve the final concentration of 20.2 mg/L of Fe (iron and manganese levels were estimated using inductivelycoupled plasma optical emission spectroscopy—ICP-OES). This M9^(+Fe)^ medium constituted the growing medium in the case of the Gram-negative rods. All forms of lactoferrin were then added to the M9^(+Fe)^ broth in order to determine the increase or decrease in the Gram-negative bacterial population numbers. For *Staphylococcus aureus*, a liquid TSB medium was used as a growing medium.

For probiotics strains *Lactobacillus and Bifidobacterium*, we chose a liquid MRS and TPY medium, respectively. The contents of the iron and manganese ions were 0.6 mg/L and 20 mg/L for MRS and 4.5 mg/L and <0.5 mg/L for TPY, respectively. In this case, the concentration of iron and manganese ions was too high to observe the effect of lactoferrin metal saturation. It was necessary to reduce these concentrations to the minimum level, thus allowing monitoring of the growth of probiotic bacteria. In order to achieve this, the above mentioned broths were incubated for 2 h with a Chelex^®^ 100 sodium form (Sigma-Aldrich, 50 g/L). An ion-exchange resin was then removed by filtration. That way, we reduced the concentration of Fe ions and Mn ions to a final value of <0.5 mg/L in the MRS and TPY broths. The various lactoferrin forms were added to such media (MRS^(−)^ or TPY^(−)^), which resulted in final media with various contents of Fe and Mn.

Detailed information on the concentration of metals are presented in Table 1.

### 2.5. Bacterial Growth Evaluation

Freshly prepared bacterial suspensions (as described above) at a density of 1 × 10^8^ colony colony-forming unit per mL (CFU/mL) were centrifuged (10 min, 6000× *g*) and washed twice with sterile phosphate-buffered saline. Dilutions in the appropriate broth were made to adjust the density to 1 × 10^5^ CFU/mL. Finally, 200 μL of this suspension were added to 1.8 mL of the appropriate medium (growing media or growing media with different lactoferrin forms) to obtain a population number of 1 × 10^4^ CFU/mL.

Three independent repetitions of every experiment were performed with three technical replicates in each repetition. We evaluated bacterial growth using two methods:

(a) Semi-quantitative assessment. Optical density (OD) monitored every 30 min by measuring the absorbance of 200 μL of the culture media at 620 nm using a Tecan Infinite M200 PRO plate reader.
(b) Quantitative assessment. After 24 h (and 48 h for Bifidobacterium) at 37 °C, bacterial cultures were seeded quantitatively on appropriate agar plates.

### 2.6. Animals

Male albino Wistar rat pups [WistarKrf (Wi) Wu)] with a 20–30 g body weight were used throughout the study. The pups stayed with the mother until 12 days post-birth when they were prematurely weaned. Animals were fed standard laboratory chow and water ad libitum. They were housed in standard cages in temperature-controlled rooms (18–22 °C, 50–60% humidity) under a 12 h light cycle (06:00–18:00) and appropriate environmental enrichment was introduced in the cages. All animal procedures performed conformed with the guidelines from Directive 2010/63/EU of the European Parliament on the protection of animals used for scientific purposes and approved by the Jagiellonian University Ethical Committee on Animal Experiments (no. 190/2012).

### 2.7. In Vivo Experimental Design

A total number of 24 male rat pups were used to study the effect of oral administration of various lactoferrin forms on the microbial community in the neonatal gut. The main focus was on population numbers of *Escherichia coli* and staphylococci as well as the probiotic bacteria of the *Lactobacillus* and *Bifidobacterium* genera. Given the typical standard deviations of CFU/g range between 0.5–0.8 log, we calculated that the usage of six animals per group would be enough to observe a significant difference between means of 1.5 log with 80% power (at a significance level of 0.05). Six dams were used and their litters were reduced to four male pups. On day 12 post-birth, rat pups were prematurely weaned and randomly assigned into four cages corresponding to experimental units/groups (every pup from each litter was placed in a different cage). On days 14–16, lactoferrin forms differing in metal saturation (namely metal-depleted apoLf, iron-saturated holoLf and manganese-saturated MnLf; water was used for a control group) were administered orally at a dose of 300 mg/kg/day (every day between 8–9 am in the treatment room). This was to mimic the process of lactoferrin supplementation in humans. On day 17, the animals were euthanized using pentobarbital in the laboratory (100 mg/kg body weight, i.p., standard procedure for euthanasia) and fecal content was extracted and plated quantitatively on selective media. The order in which the animals were treated was random as the procedures were not time-consuming and were performed quickly and efficiently. No adverse events were observed during the study.

Experimental units, that is groups given either the vehicle (control group) or lactoferrin form (experimental groups), were arranged as explained below:

- Group 1 (*n* = 6): control—weaned pups administered vehicle (water per os);
- Group 2 (*n* = 6): apoLf—weaned pups administered apolactoferrin;
- Group 3 (*n* = 6): holoLf—weaned pups administered hololactoferrin;
- Group 4 (*n* = 6): MnLf—weaned pups administered manganese-saturated lactoferrin.

### 2.8. Statistical Analysis

Herein data were presented as mean (growth curves), mean ± SEM (for colony forming unit graphs) or scatter plots (mean and value for every animal in the group for in vivo studies). The test for normality was performed using the Kolmogorov–Smirnov test. Data were analyzed using one-way ANOVA, followed if significant with a post-hoc Tukey test. The statistical analysis was performed in Graphpad Prism v. 5.01 (GraphPad Software, Inc., San Diego, CA, USA). The significance level was set at 0.05.

## 3. Results

### 3.1. The Effect of Various Forms of Lactoferrin on Pathogenic Bacterial Growth

**Figure 1:**
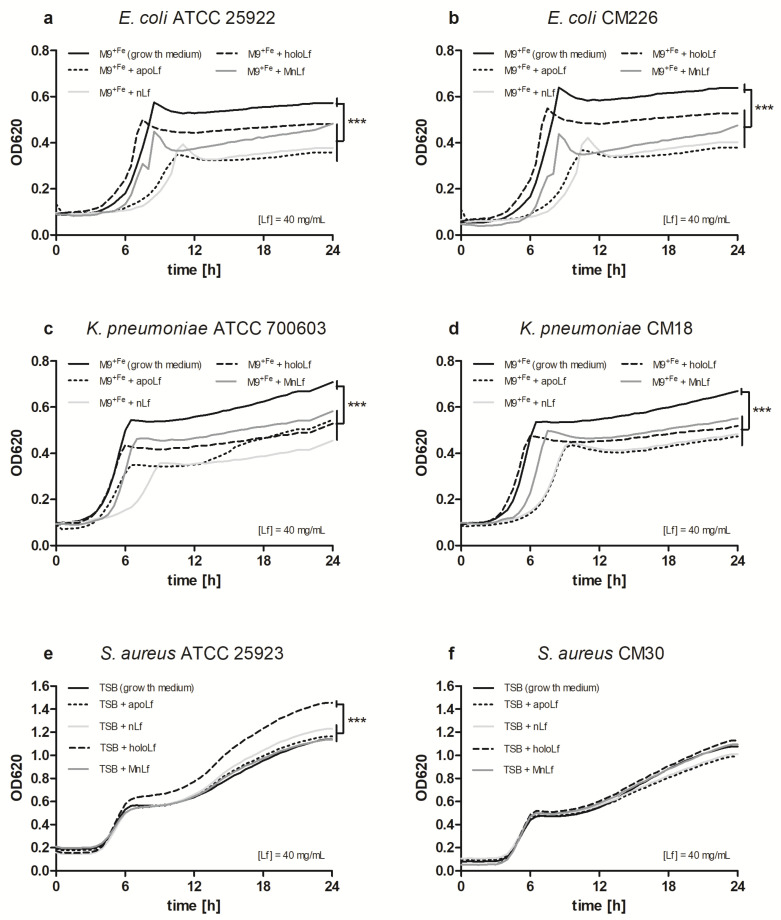
Semi-quantitative assessment of pathogenic bacterial growth upon incubation with various lactoferrin forms: *Escherichia coli* ATCC 25922 (**a**), *E. coli* CM226 (**b**), *Klebsiella pneumoniae* ATCC 700603 (**c**), *K. pneumoniae* CM18 (**d**), *Staphylococcus aureus* ATCC 25923 (**e**), *S. aureus* CM30 (**f**). Statistical significance was evaluated using one-way ANOVA, *** *p* < 0.001. Lf, lactoferrin; apoLf, apolactoferrin; holoLf, hololactoferrin; nLf, native lactoferrin; MnLf, manganese-saturated lactoferrin; TSB, tryptic soy broth.

Using the semi-quantitative assessment of pathogenic bacteria growth upon incubation with different forms of lactoferrin, several statistically significant differences were observed only for the highest used concentration of lactoferrin (the data for lower concentrations of lactoferrin forms are shown in Supplementary Materials Figure S1). For Gram-negative rods, both *E. coli* and *Klebsiella pneumoniae* reference and clinical strains behaved similarly, i.e., after 24 h we observed a statistically significant decrease in the population number in the medium with the addition of all tested forms of lactoferrin (nLf, holoLf, apoLf, MnLf) in comparison with the growing medium (Figure 1a,d).

**Figure 2:**
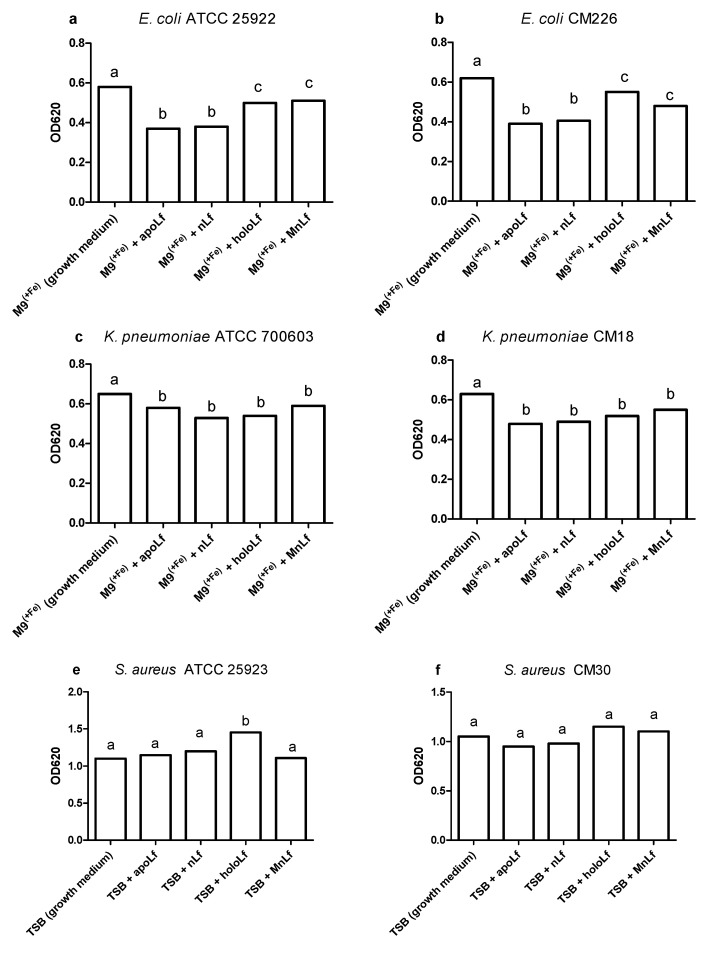
Optical density of pathogenic bacteria cultures after 24 h culture in the presence of various lactoferrin forms. *E. coli* ATCC 25922 (**a**), *E. coli* CM226 (**b**), *K. pneumoniae* ATCC 700603 (**c**), *K. pneumoniae* CM18 (**d**), *S. aureus* ATCC 25923 (**e**), *S. aureus* CM30 (**f**). All of the observed differences (bars without a common letter) were significant at *p* < 0.001 in one-way ANOVA.

Employing OD data, we compared the antibacterial activity of various forms of lactoferrin with each other (Figure 2). For both reference and clinical *E. coli* strains, the largest decrease in population was observed between the forms of lactoferrin containing the lowest amount of iron. These were apoLf and nLf, as opposed to holoLf (Fe concentration of 21.5 and 27.1 vs. 60.3 mg/L, respectively).

Moreover, the population numbers of both *E. coli* strains in holoLf were significantly lower compared with the M9^(+Fe)^ growth medium (Fe concentration of 20.2 mg/L) despite the fact that holoLf contained higher concentrations of Fe ions (60.3 mg/L). This may indicate that lactoferrin itself, regardless of the degree of ion saturation, exerted some antimicrobial effect on *E. coli*.

In case of *K. pneumoniae* (both reference and clinical strains), all tested forms of lactoferrin acted similarly. They significantly reduced the number of the *Klebsiella* population in comparison with the M9^(+Fe)^ growth medium (Figure 2c,d).

Based on OD data obtained for both reference and clinical *S. aureus* strains, we did not observe a decrease in population density (Figure 1e,f). On the contrary, solely holoLf positively impacted the growth rate of the reference *S. aureus* ATCC 25923 strain, which could imply the utilization of the iron from the holoLf (Figure 2e). No such effect was observed for the clinical strain of *S. aureus* (Figure 2f).

**Figure 3:**
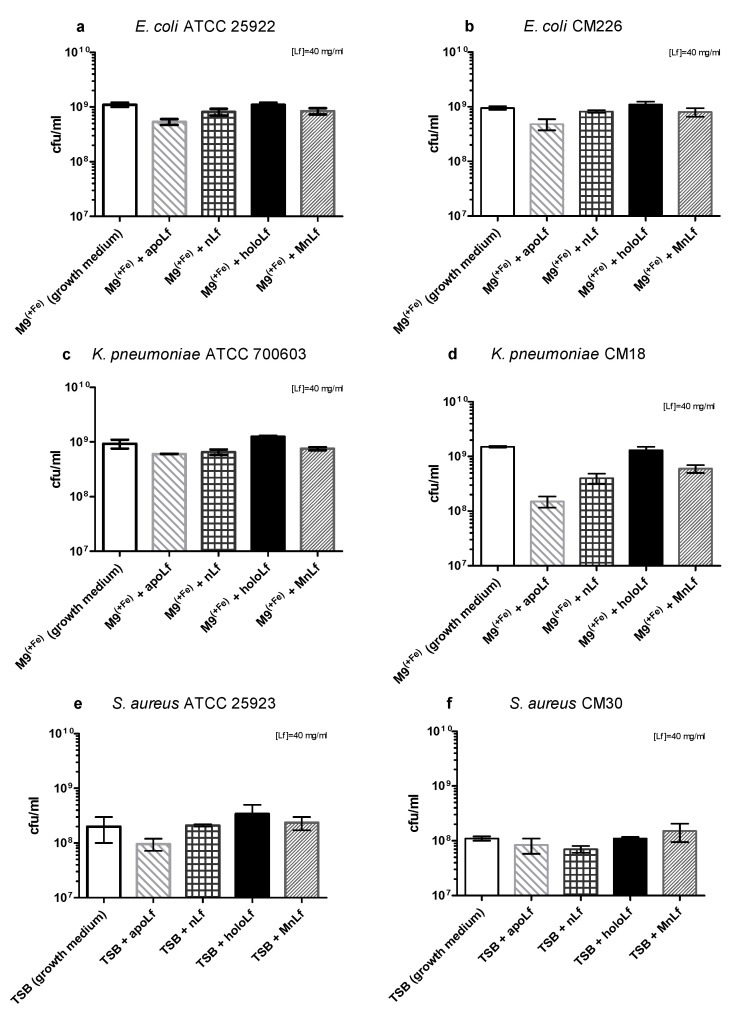
Quantitative assessment of tested pathogenic bacterial strains growth after 24 h incubation with various lactoferrin forms: *E. coli* ATCC 25922 (**a**), *E. coli* CM226 (**b**), *K. pneumoniae* ATCC 700603 (**c**), *K. pneumoniae* CM18 (**d**), *S. aureus* ATCC 25923 (**e**), *S. aureus* CM30 (**f**).

We could only observe some tendencies in the population numbers of Gram-negative rods when using a quantitative plating method (Figure 3a–d, data for lower concentrations are shown in Figure S2). The reduction of population numbers of both *E. coli* and *K. pneumoniae* strains was more efficient when apoLf was added to the liquid broth but the changes were not statistically significant. The holoLf population numbers were comparable with the growth medium whereas the native Lf and MnLf had an intermediate impact.

Similar trends were observed for the reference strain of *S. aureus* ATCC 25923; holoLf seemed to increase the population number of this strain (as compared with the growth medium, Figure 3e). This is in agreement with the results from the optical density measurements (Figure 2e). However, in the case of the clinical *S. aureus* strain, no prominent tendencies were observed when comparing the population numbers upon incubation with various lactoferrin forms (Figure 3f).

### 3.2. The Effect of Various Forms of Lactoferrin on Probiotic Bacterial Growth

**Figure 4:**
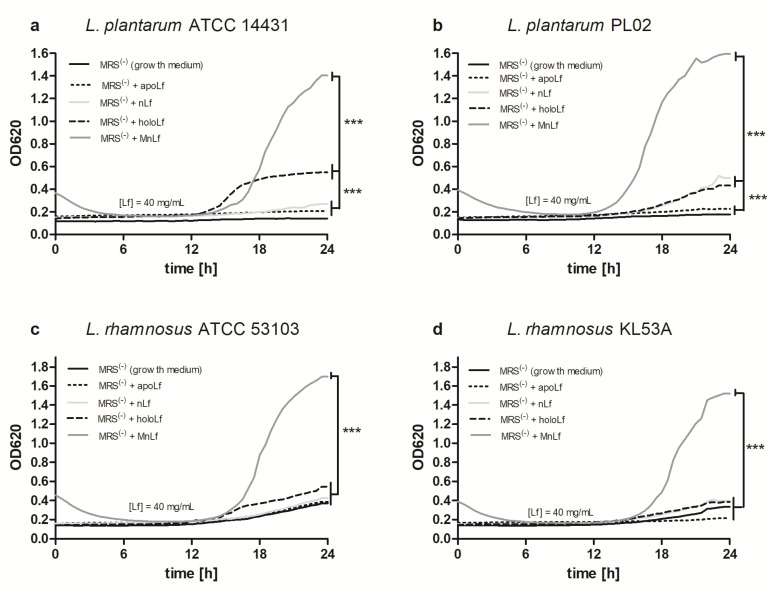
Semi-quantitative assessment of the growth of *Lactobacillus* strains upon incubation with various lactoferrin forms: *Lactobacillus plantarum* ATCC 14431 (**a**), *L. plantarum* PL02 (**b**), *Lactobacillus rhamnosus* ATCC 53103 (**c**), *L. rhamnosus* KL53A (**d**). *** *p* < 0.001 in one-way. MRS, Man, Rogosa and Sharpe broth.

**Figure 5:**
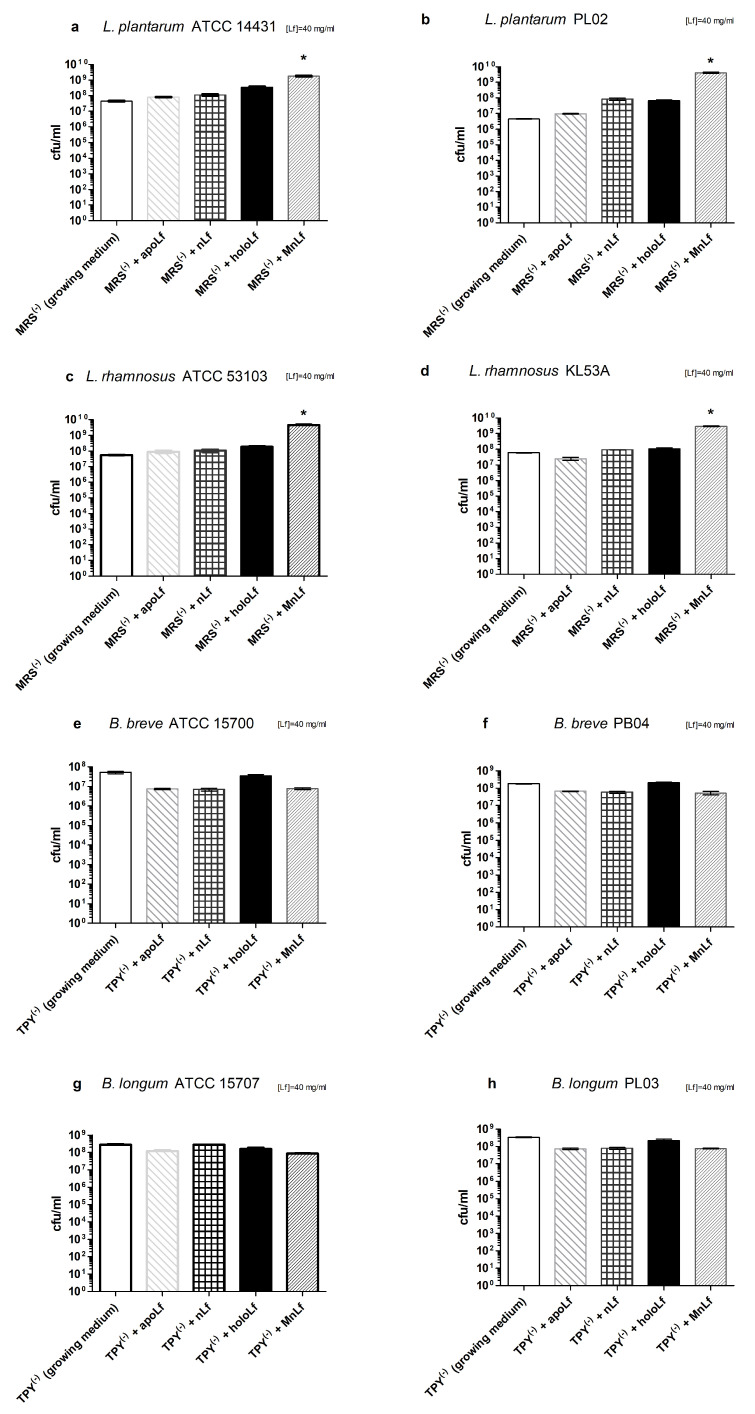
Quantitative assessment of tested strains growth after 24 h incubation with various lactoferrin forms: *L. plantarum* ATCC 14431 (**a**), *L. plantarum* PL02 (**b**), *L. rhamnosus* ATCC 53103 (**c**), *L. rhamnosus* KL53A (**d**), *Bifidobacterium breve* ATCC 15700 (**e**), *B. breve* PB04 (**f**), *Bifidobacterium longum* ATCC 15707 (**g**), *B. longum* PL03 (**h**). Statistical significance was evaluated for tested lactoferrin forms in comparison with the growth medium using one-way ANOVA, * *p* < 0.05. MRS, Man, Rogosa and Sharpe broth; TPY, trypticase-phytone-yeast broth.

Optical density measurements proved that manganese-saturated lactoferrin had a major positive impact on population numbers of all tested strains of *Lactobacillus* (Figure 4a–d). For *L. plantarum* strains, holoLf and nLf also exerted some prebiotic effect; however, it was notably lower than that observed for the manganese-saturated protein (Figure 4a,b). Furthermore, the increase in the optical density of the *Lactobacillus* culture was also observed when lower concentrations of manganese-saturated lactoferrin was used (Figure S3). Interestingly, the beneficial impact of MnLf on *Lactobacillus* strains could also be detected using a standard quantitative plating method (Figure 5a–d). For all tested *Lactobacillus* strains, the population number increased significantly upon the culture only in the presence of manganese-saturated lactoferrin (data for lower concentrations are shown in Figure S4).

The growth of strains of the *Bifidobacterium* genus was only evaluated using quantitative plating due to the strictly anaerobic requirements of these bacteria. We observed no significant changes in the population numbers of the tested strains (Figure 5e–h; lower concentrations in Figure S4).

### 3.3. Investigation of Various Lactoferrin Forms Effects In Vivo

In order to verify whether various lactoferrin forms might have a different effect on the gut microbiota we employed the animal model using the prematurely weaned rat pups. Following a three day oral supplementation with metal-depleted (apoLf), iron-saturated (holoLf) and manganese-saturated (MnLf) lactoferrin, the animals were euthanized and fecal content was homogenized and plated quantitatively. We focused on the species most relevant for the bacterial translocation, which was potentially pathogenic *Escherichia coli*. On the other hand, we quantitated the probiotic strains of the *Lactobacillus* genus, which may contribute to the restoration of gut homeostasis. It should be noted that bacteria of the *Staphylococcus* and *Bifidobacterium* genera were not present in the tested fecal samples, which could be attributed to both differences in the intestinal microbiota composition between human and rats as well as the type of feeding that the rat pups received after weaning [12].

**Figure 6:**
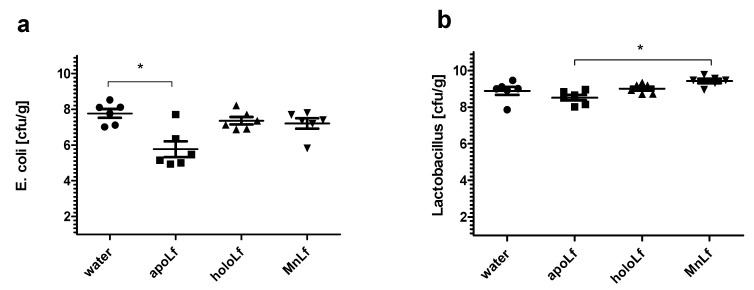
Quantitative assessment of population number of *Escherichia coli* (**a**) and lactobacilli (**b**) in fecal samples of prematurely weaned rat pups from a control group or fed with tested lactoferrin forms (*n* = 6 for each group). * *p* < 0.05 (one-way ANOVA).

We observed a significant reduction in the population numbers of *Escherichia coli* upon administration of apolactoferrin (Figure 6a) in comparison with the control group (fed with vehicle only, which was water). Other forms of lactoferrin seemed to only mildly decrease the population numbers of *E. coli* (but no significant differences were observed). These results were in concordance with the in vitro experiments in which bacterial culture OD was the lowest for the apolactoferrin group in the case of both studied *E. coli* strains (Figure 1a,b). At the same time, metal-saturated forms of lactoferrin (both holoLf and MnLf) did not exhibit much impact on the *E. coli* culture OD, which was also the case in the in vivo setup.

The population numbers of the *Lactobacillus* family did not change as remarkably (Figure 6b) as they did in vitro (Figure 5a–d). There were some tendencies showing that MnLf was the most potent in increasing the population numbers of lactobacilli (statistically significant difference vs. apolactoferrin). These results further confirmed that manganese present in lactoferrin might be a suitable source of this element for lactobacilli [13]. It seems plausible that the prebiotic properties of MnLf observed in vitro were attenuated in the animal model. These could be explained by the in vivo modification of MnLf due to pH changes across the rat gastrointestinal tract especially because our previous studies [10] reported that MnLf released manganese in the acidic environment.

The limitation of the study is the fact that the rats weaned 12 days post-partum were better equipped to fight sepsis than human newborns. Currently, we are working on developing a model in which the bacterial translocation from the intestinal lumen to the bloodstream could be induced in prematurely weaned rat pups by coexisting infection induced by the intraperitoneal administration of a *Staphylococcus haemolyticus* suspension.

## 4. Discussion

Lactoferrin, thanks to its multifunctional activities, has been recognized as one of the most important components of women’s milk. Publications dating from the 1980s to the early years of the 21st century emphasize the strong bacteriostatic and bactericidal activity of lactoferrin, especially the apolactoferrin, versus various microorganisms including *Escherichia coli* [14,15]. This has prompted the usage of lactoferrin as an innovative nutritional supplement mostly for infant formulas [16]. However, the reports published in the last few years present a much less impressive view on the bactericidal effect of lactoferrin. In our study, we examined the bactericidal effect of various forms of lactoferrin (apoLf, nLf, holoLf, MnLf) at concentrations of 0.6, 5 and 40 mg/mL because high concentrations of lactoferrins have been considered safe and used in oral dietary supplements for newborns [11].

Our studies have shown that lower concentrations of bovine lactoferrin exerted no antibacterial effect against pathogenic bacteria. Using a semi-quantitative method, after 24 h we observed a significantly decreased value of OD for Gram-negative bacteria in both *E. coli* and *K. pneumoniae* strains under the influence of all forms of lactoferrin (at 40 mg/mL) vs. the control. This may indicate that the lactoferrin was responsible for the bacteriostatic effect for Gram-negative strains (Figure 1). Moreover, we showed statistical differences in the decrease in the population size between apoLf vs. holoLf in the case of the *E. coli* strains (Figure 2a,b). This might suggest that apoLf could be more potent in limiting the bacterial translocation phenomenon.

On the contrary, this kind of difference between forms was not demonstrated for the *Klebsiella* strains. This may indicate that the antibacterial effect of iron-depleted lactoferrin (apoLf) depends on the species of Gram-negative rods and probably *E. coli* is a more potent competitor with apoLf for Fe ions of the same pool. It seems plausible that lactoferrin is engaged in a battle for iron acquisition but some pathogens are capable of counteracting such iron-binding proteins by synthesizing siderophores, small high affinity iron-chelating molecules, or through iron acquisition from other sources [17].

Based on the quantitative (CFU/mL) plating, after 24 h we did not observe any antibacterial effects of any form of lactoferrin on the population of both species of Gram-negative bacteria (Figure 2a–d).

The differences between the results received using OD and CFU methods are due to the very nature of the optical density measurement. By monitoring optical density, we detected both live and dead bacteria as well as various metabolites such as peptides, DNA or exopolysaccharides (EPS) that comprised the three-dimensional structure of bacterial biofilm. On the other hand, a quantitative culture method allowed for the quantification of only living forms of bacteria. It still constitutes the golden standard for microbiological research.

Interestingly, recently we observed a substantial shift in the literature regarding both the results and methodology of studies of Lf impact on pathogenic species. More recent reports do not show such a strong reduction of population numbers of those bacteria but only minor changes that were observed using semi-quantitative methods. We believe that such fluctuations (not exceeding one order of magnitude) might not be significant enough to affect the microbial ecology of the gut.

Based on these results, we could speculate that—only in case of Gram-negative rods—lactoferrin (at a concentration of 40 mg/mL, regardless of the metal ion saturation) shows more of an anti-biofilm than a bactericidal effect. Such activity has been demonstrated before for lactoferrin. It was shown to inhibit the biofilm formation of *Pseudomonas aeruginosa* [18]. We could theorize that the inhibition of *E. coli* and *K. pneumoniae* biofilm development by lactoferrin could facilitate the penetration of both antibiotics and reactive oxygen species into the deeper layers of the bacterial biofilm.

Furthermore, based on earlier in vitro studies, we know that lactoferrin can actively block the immunostimulatory action of endotoxin (LPS) from Gram-negative rods, which is of great importance in the case of neonatal bacteremia and endotoxemia originating from infections in the gastrointestinal tract. Perhaps, due to its high affinity for LPS, lactoferrin may also effectively bind to the structure of endotoxin in the cell wall of Gram-negative rods and thus may interfere with the proper production of bacterial biofilms.

Additionally, only the apolactoferrin form induced a significant decrease of gut *E. coli* population in an in vivo experimental setup using prematurely weaned rat pups. In comparison with the control, only the metal-depleted form of the protein administered orally led to a notable reduction of the population number of *E. coli*. This further confirms the hypothesis that apolactoferrin would be the most suitable candidate for oral supplements that could limit the overgrowth of *Enterobacteriaceae*, a particularly dangerous group of microorganisms responsible for a lower percentage of late blood infections (30–40%) and are associated with higher mortality [19,20].

In the case of *Staphylococcus* strains, we did not observe any decrease in the bacterial population numbers using either quantitative plating or semi-quantitative measurements. We may infer that, for *Staphylococcus* strains, lactoferrin did not exhibit either antibacterial or anti-biofilm effects. Therefore, it seems that lactoferrin might not be as potent as a supplement in the prevention of early-onset sepsis, which is mostly caused by Gram-positive staphylococci [21].

Interestingly, data presented here highlight the substantial role of lactoferrin saturated with manganese in the regulation of the *Lactobacillus* strain population. We observed a notable increase in population numbers of all studied strains of *L. plantarum* and *L. rhamnosus* cultured in the presence of 40 mg/mL MnLf. Additionally, the population numbers for 40 mg/mL MnLf were significantly higher than for the growth medium where we observed an increase ranging from 1.5 to 3 orders of magnitude. In the course of evolution, bacteria of *Lactobacillus* genus have become independent of iron supply by replacing iron with manganese in their protein active sites [2]. In this in vitro study, MnLf was the only source of the element in the culture media and it became a potent supply of the metal for those bacteria, resulting in a significant increase in their population numbers. This phenomenon, however, was not observed for tested *Bifidobacterium* strains. Furthermore, it was confirmed in vivo that manganese-saturated lactoferrin could enhance the *Lactobacillus* population numbers in the gut as opposed to other tested forms such as apo- and hololactoferrin. It is worth noting that such a potent positive effect on lactobacilli population numbers could translate into a major improvement of the balance in the gut microbial community achieved by concurrently limiting the growth of *Enterobacteriaceae*.

## 5. Conclusions

**Figure 7:**
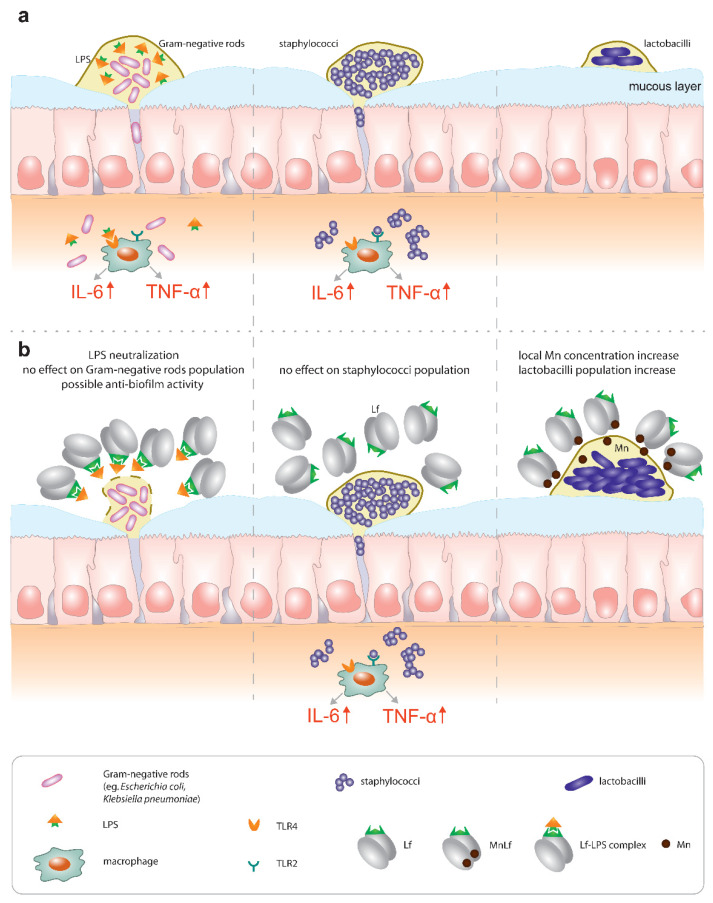
Potential activity of lactoferrin towards Gram-negative rods (left panel), staphylococci (middle panel) and lactobacilli (right panel). (**a**) In the absence of lactoferrin, both Gram-negative rods (*Escherichia, Klebsiella*) and staphylococci readily translocated through the intestinal lumen and induced an inflammatory response via toll-like receptor (TLR) signaling. Population numbers of probiotic bacteria of the Lactobacillus genus were not sufficient for gut colonization. (**b**) In the presence of lactoferrin, the population of Gram-negatives was slightly decreased potentially due to the inhibition of biofilm formation. The Staphylococci population was not affected by the presence of lactoferrin. *Lactobacillus* population numbers increased due to manganese release from MnLf.

In conclusion, our data suggest the hypothesis that lactoferrin might contribute to the regulation of the homeostasis in the gut in various ways:

1. Primarily, lactoferrin (particularly apolactoferrin) could provide mild bacteriostatic or potentially anti-biofilm forming activity towards Gram-negative rods, which was confirmed by in vivo studies. Additionally, our previous studies confirmed that Lf can counteract the proinflammatory effect of endotoxins by sequestrating them into an Lf-LPS complex and reducing the local inflammatory response in the gut [5] (Figure 7, left).
2. On the contrary, our results did not confirm any antimicrobial effect of Lf against Staphylococcus strains (Figure 7, middle). We infer that the major activity of lactoferrin does not lie in the regulation of population numbers of Gram-positive pathogenic species. We suspect that lactoferrin would not be an effective agent in the prevention of early-onset sepsis commonly caused by staphylococci.
3. Finally, a very promising activity could be seen for manganese-saturated lactoferrin as it was found to possess the ability to enhance *Lactobacillus* population numbers (Figure 7, right), which was also confirmed using the animal model.

Lactoferrin has long been implicated as a potent bioactive ingredient of dietary supplements such as infant formulae. However, little attention has been paid to both the metal saturation of lactoferrin as well as the necessity to provide a continuous supply of this protein in the neonatal gut. For instance, the endotoxin-neutralizing properties of lactoferrin require the constant presence of the protein in the gut lumen, which can only be achieved by breastfeeding on demand or oral supplementation with proper formulas. Lactoferrin seems to be relevant for the prevention of late-onset sepsis (of Gram-negative origin, associated with higher mortality) because it is capable of hindering the impact of *Enterobacteriaceae*, both directly (by limiting their growth and possibly biofilm formation) and through LPS neutralization. On the other hand, lactoferrin does not seem to counteract Gram-positive bacteria (the most common pathogens causing late-onset sepsis), which underlines the importance of proper sepsis diagnostics. Lastly, manganese-saturated lactoferrin introduces an interesting opportunity to tip the balance of the microbial community in favor of *Lactobacillus* strains that exert antibacterial properties against pathogenic species and effectively compete with them for receptor sites on the surface of intestinal epithelial cells.

Our findings confirmed in both in vitro and in vivo studies seem to correspond with the latest clinical trial results that no longer confirm the previously established effectiveness of oral lactoferrin supplementation for sepsis prevention. We suggest that the concept of omnipotent lactoferrin should be revised. Further studies are required to either confirm or deny its properties and verify whether its activity is dependent upon metal saturation. Our next studies will focus on evaluating the role of various lactoferrin forms in supplementation in the rat model of bacterial translocation.

## Figures and Tables

**Table 1:** Liquid culture media used in studies on bacterial growth.

*Gram-negative rods:* *Escherichia coli, Klebsiella pneumoniae*	Growth Medium	Fe Concentration (mg/L)	Mn Concentration (mg/L)
	M9^(+Fe)^	20.2	<0.5
	Growth medium with addition of different forms of lactoferrin (40 mg/mL)	Fe concentration (mg/L)	Mn concentration (mg/L)
	M9^(+Fe)^ + apoLf	21.5	<0.5
	M9^(+Fe)^ + nLf	27.1	<0.5
	M9^(+Fe)^ + holoLf	60.3	<0.5
	M9^(+Fe)^ + MnLf	22.1	26.8
*Gram-positive coccus:* *Staphylococcus aureus*	Growth medium	Fe concentration (mg/L)	Mn concentration (mg/L)
	TSB	1.0	<0.5
	Growth medium with addition of different forms of Lactoferrin	Fe concentration (mg/L)	Mn concentration (mg/L)
	TSB + apoLf	2.2	<0.5
	TSB + nLf	6.7	<0.5
	TSB + holoLf	42.9	<0.5
	TSB + MnLf	2.4	27.2
*Probiotics strain:* *Lactobacillus*	Growth medium	Fe concentration (mg/L)	Mn concentration (mg/L)
	MRS^(−)^	<0.5	<0.5
	Growth medium with addition of different forms of lactoferrin	Fe concentration (mg/L)	Mn concentration (mg/L)
	MRS^(−)^ + apoLf	1.5	1.0
	MRS^(−)^ + nLf	6.2	1.1
	MRS^(−)^ + holoLf	41.6	<0.5
	MRS^(−)^ + MnLf	1.7	27.6
*Probiotics strain:* *Bifidobacterium*	Growth medium	Fe concentration (mg/L)	Mn concentration (mg/L)
	TPY^(−)^	<0.5	<0.5
	Growth medium with addition of different forms of lactoferrin	Fe concentration (mg/L)	Mn concentration (mg/L)
	TPY^(−)^ + apoLf	1.6	<0.5
	TPY^(−)^ + nLf	6.8	<0.5
	TPY^(−)^ + holoLf	41.9	<0.5
	TPY^(−)^ + MnLf	1.4	27.1

apoLf, apolactoferrin; holoLf, hololactoferrin; nLf, native lactoferrin; MnLf, manganese-saturated lactoferrin; TSB, tryptic soy broth; MRS, Man, Rogosa and Sharpe broth; TPY, trypticase-phytone-yeast broth.

## Supplementary Materials

The following are available online at https://www.mdpi.com/2072-6643/12/11/3340/s1, Figure S1: Semi-quantitative assessment of pathogenic bacteria growth upon incubation with various lactoferrin forms added at a concentration of 0.6 or 5 mg/mL to growth medium (M9^+Fe^ or TSB): *E. coli* ATCC 25922 (**a**), *E. coli* CM226 (**b**), *K. pneumoniae* ATCC 700603 (**c**), *K. pneumoniae* CM18 (**d**), *S. aureus* ATCC 25923 (**e**), *S. aureus* CM30 (**f**). *** denotes statistically significant differences vs growth medium at *p* < 0.001 in one-way ANOVA., Figure S2: Quantitative assessment of tested pathogenic bacterial strains growth after 24 h incubation with various lactoferrin forms added at concentrations of 0.6 and 5 mg/mL to growth medium (M9^+Fe^ or TSB): *E. coli* ATCC 25922 (**a**), *E. coli* CM226 (**b**), *K. pneumoniae* ATCC 700603 (**c**), *K. pneumoniae* CM18 (**d**), *S. aureus* ATCC 25923 (**e**), *S. aureus* CM30 (**f**). Figure S3: Semi-quantitative assessment of Lactobacillus strains growth upon incubation with various lactoferrin forms added at a concentration of 0.6 or 5 mg/mL to growth medium (MRS(-)): *L. plantarum* ATCC 14431 (**a**), *L. plantarum* PL02 (**b**), *L. rhamnosus* ATCC 53103 (**c**), *L. rhamnosus* KL53A (**d**). *** denotes statistically significant differences vs growth medium at *p* < 0.001 in one-way ANOVA. FigureS4: Quantitative assessment of tested strains growth after 24 h incubation with various lactoferrin forms at concentrations of 0.6 and 5 mg/mL in appropriate growth medium (MRS^(−)^ or TPY^(−)^): *L. plantarum* ATCC 14431 (**a**), *L. plantarum* PL02 (**b**), *L. rhamnosus* ATCC 53103 (**c**), *L. rhamnosus* KL53A (**d**), *B. breve* ATCC 15700 (**e**), *B. breve* PB04 (**f**), *B. longum* ATCC 15707 (**g**), *B. longum* PL03 (**h**).
