# Supplementary material for: Lactoferrin Metal Saturation—Which Form Is the Best for Neonatal Nutrition?
Source: Nutrients. 2020 Oct 30;12(11):3340. doi: 10.3390/nu12113340 (PMC7692973; doi:10.3390/nu12113340)
Supplement: Supplementary file 1 [file nutrients-12-03340-s001.pdf]

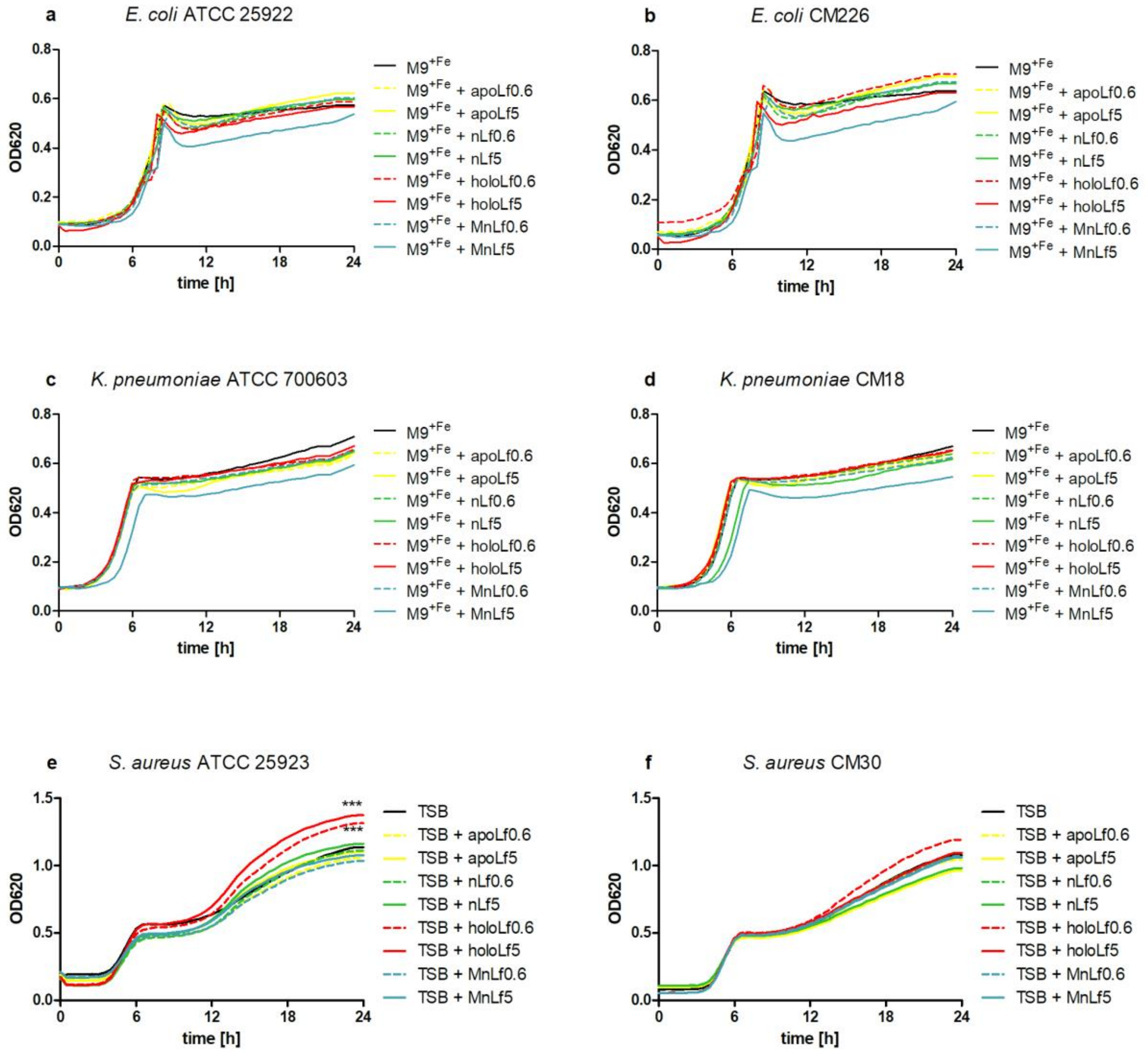


**Figure S1.** Semi-quantitative assessment of pathogenic bacteria growth upon incubation with various lactoferrin forms added at a concentration of 0.6 or 5 mg/ml to growth medium ($M9^{+Fe}$ or TSB): *E. coli* ATCC 25922 (**a**), *E. coli* CM226 (**b**), *K. pneumoniae* ATCC 700603 (**c**), *K. pneumoniae* CM18 (**d**), *S. aureus* ATCC 25923 (**e**), *S. aureus* CM30 (**f**). *** denotes statistically significant differences vs growth medium at $p<0.001$ in one-way ANOVA.

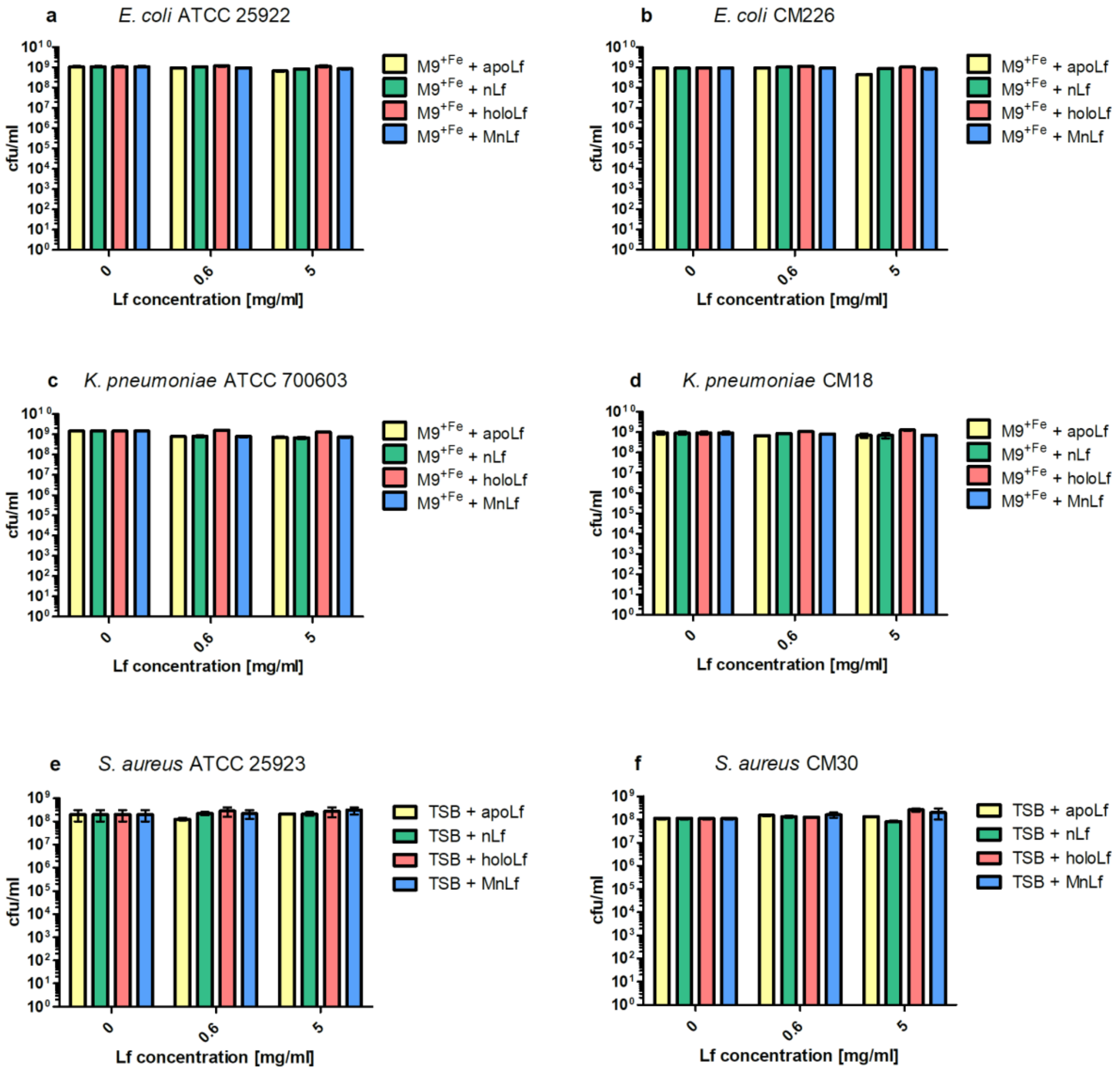


**Figure S2.** Quantitative assessment of tested pathogenic bacterial strains growth after 24 h incubation with various lactoferrin forms added at concentrations of 0.6 and 5 mg/ml to growth medium ($M9^{+Fe}$ or TSB): *E. coli* ATCC 25922 (**a**), *E. coli* CM226 (**b**), *K. pneumoniae* ATCC 700603 (**c**), *K. pneumoniae* CM18 (**d**), *S. aureus* ATCC 25923 (**e**), *S. aureus* CM30 (**f**).

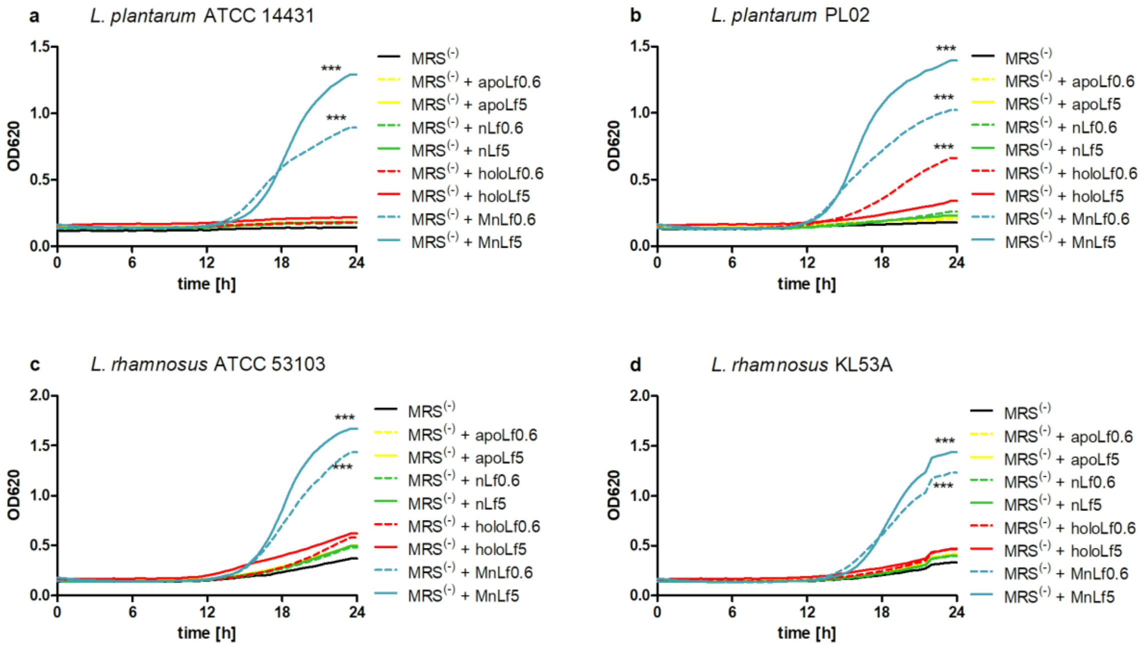


**Figure S3.** Semi-quantitative assessment of *Lactobacillus* strains growth upon incubation with various lactoferrin forms added at a concentration of 0.6 or 5 mg/ml to growth medium (MRS$^{(-)}$): *L. plantarum* ATCC 14431 (**a**), *L. plantarum* PL02 (**b**), *L. rhamnosus* ATCC 53103 (**c**), *L. rhamnosus* KL53A (**d**). *** denotes statistically significant differences vs growth medium at p<0.001 in one-way ANOVA.

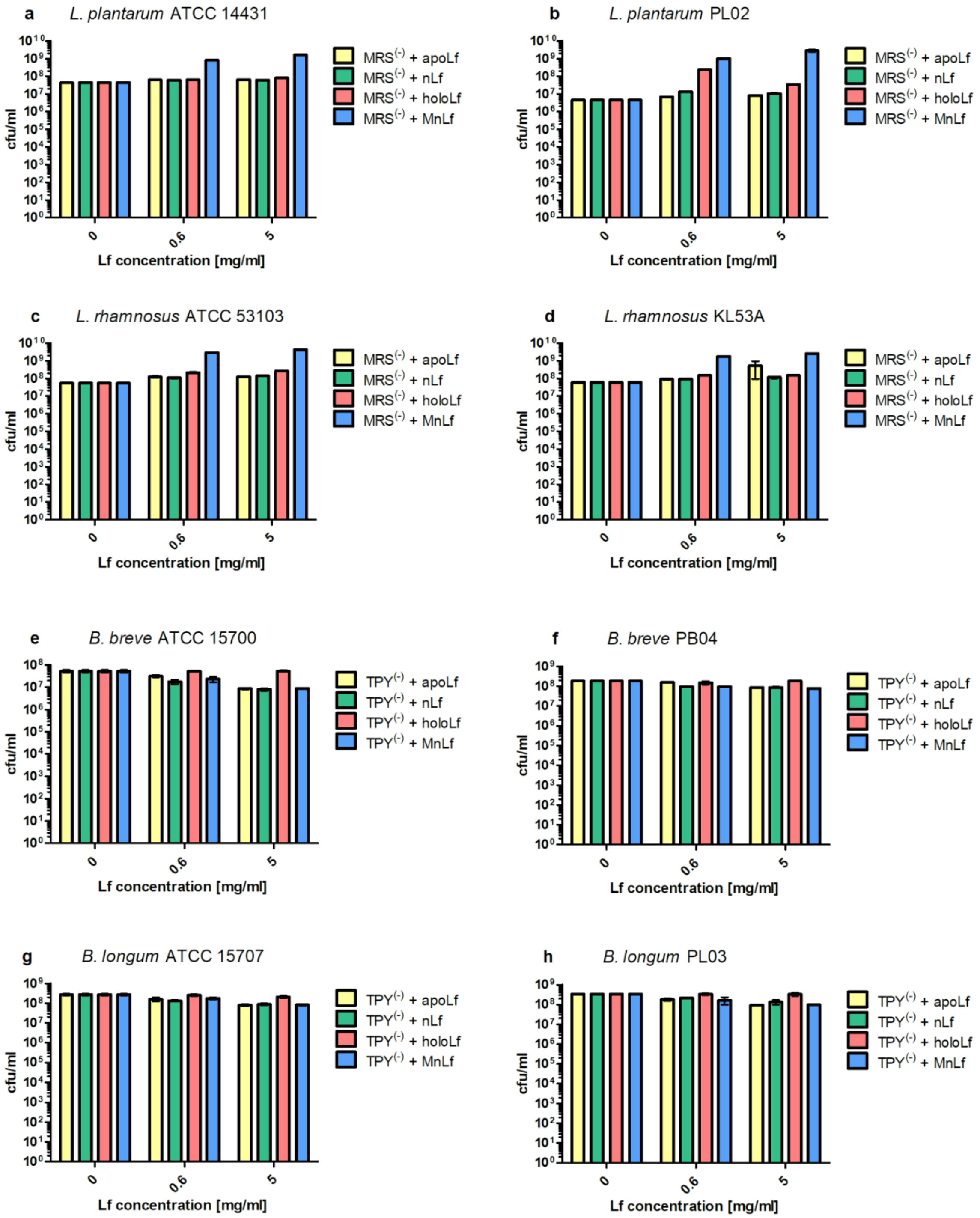


**Figure S4.** Quantitative assessment of tested strains growth after 24 h incubation with various lactoferrin forms at concentrations of 0.6 and 5 mg/ml in appropriate growth medium (MRS$^{(-)}$ or TPY$^{(-)}$): *L. plantarum* ATCC 14431 (**a**), *L. plantarum* PL02 (**b**), *L. rhamnosus* ATCC 53103 (**c**), *L. rhamnosus* KL53A (**d**), *B. breve* ATCC 15700 (**e**), *B. breve* PB04 (**f**), *B. longum* ATCC 15707 (**g**), *B. longum* PL03 (**h**).
